# The Evolving and Expanding Role of Personal Support Workers in End-of-Life Care in the Long-Term Care Setting

**DOI:** 10.1093/geroni/igaf122.1159

**Published:** 2025-12-31

**Authors:** Danielle Just, Hannah O’Rourke, Whitney Berta, Lisa Cranley

**Affiliations:** University of Toronto, Toronto, Ontario, Canada; University of Alberta, Edmonton, Alberta, Canada; University of Toronto, Toronto, Ontario, Canada; University of Toronto, Toronto, Ontario, Canada

## Abstract

**Background:**

Personal Support Workers (PSWs) play a vital role in long-term care (LTC). Initially, their role was focused on assisting residents with activities of daily living. However, as resident care needs become increasingly complex due to a rapidly aging population, PSWs’ roles have expanded. End-of-life care is now a core aspect of their responsibilities. Despite this, no formal or consistent definition of PSWs’ role in end-of-life care exists.

**Purpose:**

To explore and describe the role of PSWs in providing end-of-life care in LTC.

**Methods:**

A qualitative single case study was conducted using virtual data collection methods. Data sources included study site characteristics, documentation data, archival records, demographic data, interviews, and observations of physical artifacts.

**Results:**

Sixteen participants, including residents, family members, and LTC staff, contributed to the study. Findings revealed that PSWs frequently engage in extra-role behaviors (i.e., tasks beyond their job descriptions) when providing end-of-life care. These behaviors were widely recognized and perceived as essential for improving care quality. Additionally, the strong familial relationships between PSWs and residents were key motivators for their engagement in extra-role behaviors.

**Conclusions:**

This study underscores PSWs’ evolving and expanding role in end-of-life care and highlights their indispensable contributions to LTC. By formally recognizing and integrating PSWs’ end-of-life care responsibilities into policies, training, and workforce planning, healthcare leaders can enhance the quality of care for residents while fostering a more supportive and sustainable work environment for PSWs. This change is critical for addressing workforce shortages and ensuring high-quality end-of-life care in the LTC setting.

